# Transferability of radiomic signatures from experimental to human interstitial lung disease

**DOI:** 10.3389/fmed.2022.988927

**Published:** 2022-11-17

**Authors:** Hubert S. Gabryś, Janine Gote-Schniering, Matthias Brunner, Marta Bogowicz, Christian Blüthgen, Thomas Frauenfelder, Matthias Guckenberger, Britta Maurer, Stephanie Tanadini-Lang

**Affiliations:** ^1^Department of Radiation Oncology, University Hospital Zurich, Zurich, Switzerland; ^2^Comprehensive Pneumology Center, Institute of Lung Health and Immunity, Helmholtz Zentrum München, Member of the German Center for Lung Research (DZL), Munich, Germany; ^3^Department of Rheumatology and Immunology, University Hospital Bern, University of Bern, Bern, Switzerland; ^4^Institute of Diagnostic and Interventional Radiology, University Hospital Zurich, Zurich, Switzerland

**Keywords:** radiomics, preclinical imaging, interstitial lung disease, lung fibrosis, systemic sclerosis, bleomycin

## Abstract

**Background:**

Interstitial lung disease (ILD) defines a group of parenchymal lung disorders, characterized by fibrosis as their common final pathophysiological stage. To improve diagnosis and treatment of ILD, there is a need for repetitive non-invasive characterization of lung tissue by quantitative parameters. In this study, we investigated whether CT image patterns found in mice with bleomycin induced lung fibrosis can be translated as prognostic factors to human patients diagnosed with ILD.

**Methods:**

Bleomycin was used to induce lung fibrosis in mice (n_control = 36, n_experimental = 55). The patient cohort consisted of 98 systemic sclerosis (SSc) patients (n_ILD = 65). Radiomic features (n_histogram = 17, n_texture = 137) were extracted from microCT (mice) and HRCT (patients) images. Predictive performance of the models was evaluated with the area under the receiver-operating characteristic curve (AUC). First, predictive performance of individual features was examined and compared between murine and patient data sets. Second, multivariate models predicting ILD were trained on murine data and tested on patient data. Additionally, the models were reoptimized on patient data to reduce the influence of the domain shift on the performance scores.

**Results:**

Predictive power of individual features in terms of AUC was highly correlated between mice and patients (*r* = 0.86). A model based only on mean image intensity in the lung scored AUC = 0.921 ± 0.048 in mice and AUC = 0.774 (CI95% 0.677-0.859) in patients. The best radiomic model based on three radiomic features scored AUC = 0.994 ± 0.013 in mice and validated with AUC = 0.832 (CI95% 0.745-0.907) in patients. However, reoptimization of the model weights in the patient cohort allowed to increase the model’s performance to AUC = 0.912 ± 0.058.

**Conclusion:**

Radiomic signatures of experimental ILD derived from microCT scans translated to HRCT of humans with SSc-ILD. We showed that the experimental model of BLM-induced ILD is a promising system to test radiomic models for later application and validation in human cohorts.

## Introduction

Interstitial lung disease (ILD) defines a group of chronic, etiologically different parenchymal lung disorders, characterized by fibrosis as their common final pathophysiological stage. The prognosis of the most prevalent and severe subtypes, idiopathic pulmonary fibrosis (IPF) and ILD associated with the autoimmune disease systemic sclerosis (SSc), is as poor as that of untreated oncologic diseases (1, 2). Globally, non-malignant lung diseases including ILD rank third on the mortality scale (3).

Experimental models of fibrosing ILD are paramount for the identification of cellular and molecular key drivers of disease and as preclinical test systems for novel targeted drugs (4). The preferred and best characterized preclinical model of ILD is the murine model of bleomycin-induced lung fibrosis, which reflects important features of human ILD such as apoptosis of epithelial cells, influx of inflammatory cells into the interstitium, followed by activation of fibroblasts with increased deposition of extracellular matrix (ECM) proteins (5, 6).

Conventional endpoint measures of lung fibrosis involve histological and biochemical analyses, which, however, have certain disadvantages. To recapitulate the dynamic process of fibrosing ILD at multiple time points and to account for the high interindividual variability, large numbers of animals are required to reach significant statistical power (7). Additionally, lung biopsies are only rarely performed in human ILD (8, 9) and biopsy may not be representative for the whole lung pathology. Upcoming alternative outcome measures for translational ILD research include imaging methodologies. An integral part of the routine clinical management is medical imaging, particularly high-resolution computed tomography (HRCT), which allows non-invasive, highly sensitive, time- and spatially resolved visualization of the entire lung changes (10) and a correlative estimation of lung function (11). Similarly, in preclinical models of ILD, small animal microCT is increasingly recognized as a valuable assessment tool (4, 7). In the model of bleomycin-induced experimental ILD, the relative comparability of both imaging and molecular changes with human ILD (5, 12–15) support its suitability for translational ILD research.

The need for innovative, directly transferable, and readily applicable readouts in ILD have prompted the herein presented translational study on the potential value of the model of bleomycin-induced lung fibrosis as experimental “radiomic toolbox” for human ILD. Radiomics is a powerful strategy for in-depth analysis of pathologic tissue phenotypes by computational extraction of quantitative imaging features from medical images (16, 17). Radiomic features provide objective information on tissue shape, intensity, and texture on a molecular scale as demonstrated by studies on tumor biology showing correlation with tissue-based genomics and proteomics data (18–21). As image-derived tissue surrogates, their potential use as virtual biopsies could make radiomics analyzes an ideal tool for clinical decision support in ILD especially since radiomic features have also been shown to predict disease outcome and response to therapy (18, 19, 22–25). However, compared with oncology (18, 20–22), research into the potential of radiomics in non-malignant lung diseases is limited (26–30).

Nevertheless, the available literature on human lung pathologies, including chronic obstructive pulmonary disease, radiation-induced pneumonitis and connective tissue disease-related ILD showed that texture-based analysis of CT images can be superior compared to the visual or histogram-based measures for diagnosis (28, 31, 32). Few studies investigated the use of radiomics in experimental settings. Eresen et al. used MRI radiomics for prediction of response to vaccine therapy in a mouse model of pancreatic ductal adenocarcinoma (33, 34). Nunez et al. analyzed suitability of MRI radiomics for diagnosis of preclinical GL261 glioblastoma (35). Other researchers focused on radiomic-based prediction of liver metastases or liver fibrosis in mice (36, 37).

To date no study has shown the value of animal models in radiomics research. We are not aware of any studies reporting transferability of radiomic patterns from experimental model to clinical setting. Establishing a link between preclinical and clinical radiomic patterns could enormously facilitate testing a vast range of hypotheses in an experimental setting. Such a link is currently missing. In this analysis, we evaluate if radiomic features and models can be translated from experimental to human ILD.

## Materials and methods

### Study design and data sets

**FIGURE 1:**
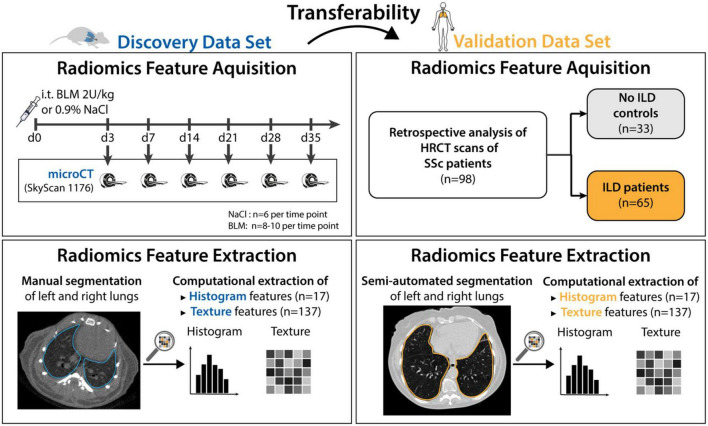
Study design. The mice data set (*n* = 91) was used to discover radiomic patterns predictive of ILD. The discovered patterns were tested in the human validation data set (*n* = 98). 55 mice were given Bleomycin to induce ILD, whereas 36 mice were given NaCl and served as the control group. The mice were euthanized at day 3, 7, 14, 21, 28, and 35 and scanned with a microCT scanner. Afterward, classification models were trained to predict occurrence of ILD based on images acquired from the scanner. The 98 patients from the validation data set were retrospectively collected. All patients were scanned with HRCT and graded according to the Goh scale of pulmonary fibrosis. The radiomic models built using mice data were tested in patients.

Details of the study design and data sets are shown in Figure 1. In short, we investigated whether radiomic patterns indicative of ILD in mice were also present in human disease.

The preclinical model of bleomycin (BLM)-induced lung fibrosis was used to mimic human ILD. The experimental cohort consisted of 91 8-week-old female mice (C57BL/6J-rj, Janvier Labs). ILD was induced in 55 mice via intratracheal instillation of bleomycin (2 U/kg; Baxter 15,000 I.U.) as described in (6, 14, 38). The 36 control animals received equivalent volumes of 0.9% NaCl solution. Mice were randomized into the different experimental groups and instillation was performed blinded. Pulmonary micoCT scans were performed at different days (days 3, 7, 14, 21, 28, and 35) after bleomycin instillation to reflect different disease stages. Different mice were scanned at every time point as the animals were euthanized after image acquisition. Scanning mice at different time points after fibrosis induction did not serve a particular purpose in this work. Such design was chosen because this experimental data was also used in other studies which examined temporal aspect of fibrotic development.

A cohort of 98 SSc patients being followed at the Department of Rheumatology, University Hospital Zurich represented the validation data set. All included patients met the following criteria: diagnosis of SSc according to the Very Early Diagnosis of Systemic Sclerosis (VEDOSS) (39) or the 2013 American College of Rheumatology//European League against Rheumatism (ACR/EULAR) classification criteria (40), and availability of an HRCT scan. Patient characteristics are provided in Table 1.

**TABLE 1:** Summary of patient’s demographics and clinical baseline characteristics.

Characteristics	Zurich Cohort (*n* = 98)
Age (year)	60.0 ± 19.0
**Sex**	
Male	21 (21.4%)
Female	77 (78.6%)
Disease duration (year)*	5.0 ± 8.6
**SSc subset (LeRoy 1988)**	
Limited cutaneous SSc	41 (41.8%)
Diffuse cutaneous SSc	37 (37.8%)
No skin involvement	20 (20.4%)
**Skin involvement**	
Limited cutaneous	34 (34.7%)
Diffuse cutaneous	36 (36.7%)
No skin involvement	23 (23.5%)
Only sclerodactyly	5 (5.1%)
**Autoantibodies**	
Anti-centromere positive	26 (26.5%)
Anti-topoisomerase I positive	35 (35.7%)
Anti-RNA polymerase III positive	8 (8.2%)
Anti-PMScl positive	15 (15.3%)
FVC (% predicted)	91.0 ± 37.0
DLCO (% predicted)	70.0 ± 35.0
FEV1 (% predicted)	92.0 ± 27.0
Pulmonary hypertension^*☨*^	18 (18.4%)
PAPsys (MmHg)	24.5 ± 9.8
6 min walk distance (m)	530.0 ± 172.5
SpO_2_ before 6-MWT (%)	97.0 ± 1.0
SpO_2_ after 6-MWT (%)	95.0 ± 6.8
Borg scale (unit)	3.0 ± 2.0
**Extent of lung fibrosis on CT**	
None	33 (33.7%)
Present	65 (66.3%)
Ground glass opacification	25 (25.5%)
Reticular changes	64 (65.3%)
Tractions	38 (38.8%)
Honeycombing	21 (21.4%)
Bullae	3 (3.1%)
**Radiological subtype^#^**	
NSIP	55 (56.1%)
UIP	9 (9.2%)
DIP	1 (1.0%)
Immunomodulatory therapy^§^	42 (42.9%)

Continuous variables are described as median ± interquartile range and categorical variables are present as absolute numbers with relative frequencies (percent).

*Disease duration of SSc was calculated as the difference between the date of baseline CT and the date of manifestation of the first non-Raynaud’s symptom.

^*☨*^Pulmonary hypertension was assessed by echocardiography or right heart catheterization.

^#^Radiological subtypes were only determined for SSc patients with ILD.

^§^Immunomodulatory therapy included prednisone, methotrexate, rituximab, cyclophosphamide, mycophenolate mofetil, hydroxychloroquine, tocilizumab, imatinib, azathioprine, adalimumab, leflunomid, cyclosporine.

PAPsys, systolic pulmonary artery pressure; FVC, forced vital capacity; FEV1, forced expiratory volume in 1 second; DLCO, diffusing capacity for carbon monoxide; 6-MWT, 6-min walk test; UIP, usual interstitial pneumonia; NSIP, non-specific interstitial pneumonia; DIP, diffuse interstitial pneumonia.

**FIGURE 2:**
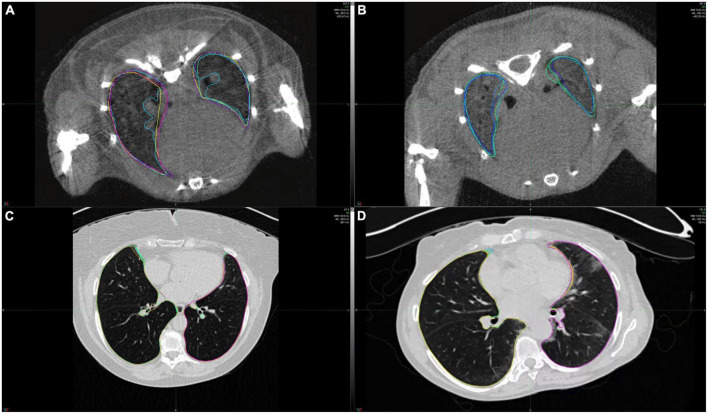
Example CT scans of healthy lungs and lungs affected with lung fibrosis. **(A)** microCT image of a healthy mice lung, **(B)** microCT image of a mice lung with lung fibrosis, **(C)** HRCT image of a healthy human lung, **(D)** HRCT image of a human lung with lung fibrosis. Lung contours marked in different colors show the extent of intra- and interobserver variability in lung segmentations for these two cases.

The extent of lung fibrosis was defined as presence of reticular changes or honeycombing within whole lung volume (Figure 2). All visual analyses were performed by a senior radiologist (TF) using a standard picture archiving and communication system workstation (Impax, Version 6.5.5.1033; Agfa-Gevaert) and a high-definition liquid crystal display monitor (BARCO; Medical Imaging Systems).

### Imaging and extraction of radiomic features

Pulmonary microCT scans were acquired in free-breathing mice with prospective respiratory gating using Bruker SkyScan 1176. The following scan parameters were used: tube voltage 50 kV, tube current 500 μA, filter Al 0.5 mm, averaging (frames) 3, rotation step 0.7 degrees, sync with event 50 ms, X-ray tube rotation 360 degrees, resolution 35 μm, and slice thickness 35 μm. Images were reconstructed with NRecon reconstruction software (v.1.7.4.6; Bruker) using the built-in filtered back projection Feldkamp algorithm and applying misalignment compensation, ring artifact reduction, and a beam hardening correction of 10% to the images.

HRCT scans were acquired using Siemens scanners (SOMATOM Definition AS, SOMATOM Definition Flash, SOMATOM Force, SOMATOM Sensation 64, SOMATOM Sensation 16, Biograph 64, LightSpeed Pro 16, LightSpeed VCT). The scans were acquired in an inspiration (breath hold) mode. The median slice thickness was 1 mm (range 0.6-2 mm) and the median tube voltage was 120 kVp (range 80-150 kVp). The reconstruction kernels included B60f, B70f, and Bl64.

The contouring of whole lungs was performed manually in mice and semi-automatically in patients (region growing algorithm followed by manual correction) by two experienced examiners (JS and MB). Left and right lungs were contoured independently and then both contours were merged to generate a single contour including both lungs.

Feature extraction from CT images was performed with Z-Rad, an IBSI-compliant (41), in-house developed Python software. CT scans of mice and patients were interpolated to an isotropic resolution of 0.15 mm and 2.75 mm, respectively. The interpolation resolutions were chosen to achieve similar ratio of voxel size to average lung volume in mice and patients. The region of interest (ROI) for feature extraction was defined as the right and the left lung considered as a single organ. Only intensity values within the range from −1,000 HU to 200 HU were considered. We used a fixed bin size of 50 HU. The radiomic features describing image intensity (histogram, *n* = 17) and texture (*n* = 137) were extracted for each mouse and patient. The texture features were based on gray level co-occurrence matrix (GLCM, *n* = 26), gray level run length matrix (GLRLM, *n* = 16), gray level distance zone matrix (GLDZM, *n* = 16), gray level size zone matrix (GLSZM, *n* = 16), neighboring gray level dependence matrix (NGLDM, *n* = 16), and neighborhood gray tone difference matrix (NGTDM, *n* = 5) to capture wide variety of intensity patterns. Additionally, GLCM and GLRLM features were extracted with two different feature aggregation methods - with and without merging. In total, 154 features were extracted. The list of radiomic features is provided in the supplement.

### Statistical analysis

For every radiomic feature, robustness against intra- and interobserver variability was examined. This was realized with estimation of the corresponding intraclass correlation coefficients (ICC). Specifically, we used consistency of ICC (1, 3) according to the Shorut and Fleiss naming convention (42). Features with ICC ≥0.75 for intra- and interobserver settings in both mice and humans were considered stable and were retained. The rest of the features were excluded from further analysis.

Univariate predictive power of the radiomic features was evaluated by estimation of the area under the receiver operating characteristic curve (AUC). To facilitate comparison of the AUC values between mice and patient data sets, we adopted a convention that AUC is equal to the probability that a radiomic feature value of a randomly chosen patient from the positive group is greater than the value of a randomly chosen patient from the negative group. This allowed us to distinguish between features that were characterized by comparable predictive power but a different *direction* of the effect, for example, AUC = 0.3 in mice and AUC = 0.7 in patients. The linear association of the AUC scores between mice and patient groups has been evaluated with Pearson correlation coefficient.

Three model architectures were considered for evaluation of model transferability from mice to patients: (1) a model based on mean image intensity (MEAN), (2) a model based on first four moments of intensity distribution (mean, standard deviation, skewness, and kurtosis; MSSK), and (3) a machine learning model based on logistic regression (ML). While the first two models are based on predefined radiomic features, the machine learning model employed embedded feature selection methods. All models were built on the mice data and were validated in the patient data.

Feature selection and model tuning was realized within 4-times repeated 5-fold cross-validation. The first step of the feature selection procedure was dimensionality reduction by removing features that were highly linearly correlated (Pearson’s *r*). The correlation threshold was one of tunable hyperparameters. The second step of feature selection was fitting a model and selection of most important features from this model which were then fed to the final classifier. In the case of a logistic regression model, the feature selection was realized with another logistic regression. In the case of, extra-trees model, most important features were extracted from a gradient tree-boosting model. The number of extracted features in both cases was one of tunable hyperparameters. For model tuning, we used 500 randomized hyperparameter samples. The optimized models were validated in patients. Additionally, the models were re-optimized in patients to evaluate transferability and predictive power of the discovered radiomic signatures rather than the models themselves. Furthermore, this allowed to reduce the influence of covariate shift between the data sets.

For visualization, statistical analysis, model building, and model testing, the following open-source Python packages were used: Matplotlib (43), NumPy & SciPy (44), Pandas (45), and scikit-learn (46).

## Results

### Influence of intra- and interobserver delineation variability on radiomic features

**FIGURE 3:**
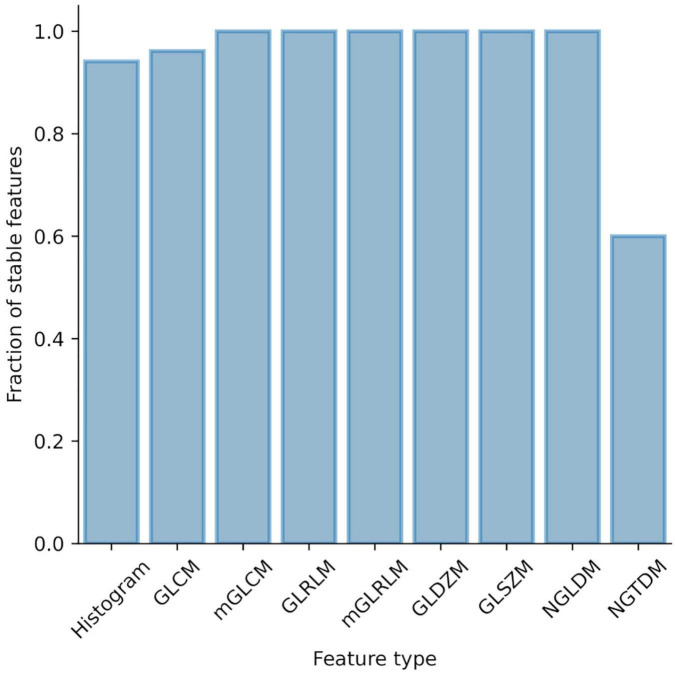
Influence of intra- and interobserver delineation variability on radiomic features stability. Proportion of unstable features stratified by feature type.

Intra- and interobserver delineation variability were evaluated separately in mice and patient data sets using 15 randomly selected cases per data set. Intraobserver variability was assessed based on delineations done by JS. Interobserver variability was assessed based on delineations provided by JS, CB, and MBr. Figure 3 shows the proportion of the unstable features per feature class. In mice, 7 features from the initial set of 154 were considered unstable (ICC < 0.75) and were excluded from the further analysis. In patients, all features were stable (ICC ≥ 0.75) so no further features were excluded.

### Discriminative power of radiomic features is highly correlated between mice and patient data

The next steps in our analysis were the investigation of univariate discriminative power of radiomic features and the correlation of AUC scores between mice and patients. ICC analysis was performed to compare two feature aggregation methods of GLCM and GLRLM features. As both feature aggregation methods rendered highly correlated results (ICC_GLCM_ = 0.99, ICC_GLRLM_ = 0.83), only one feature aggregation per feature class method was kept for further analysis to reduce feature redundancy.

**FIGURE 4:**
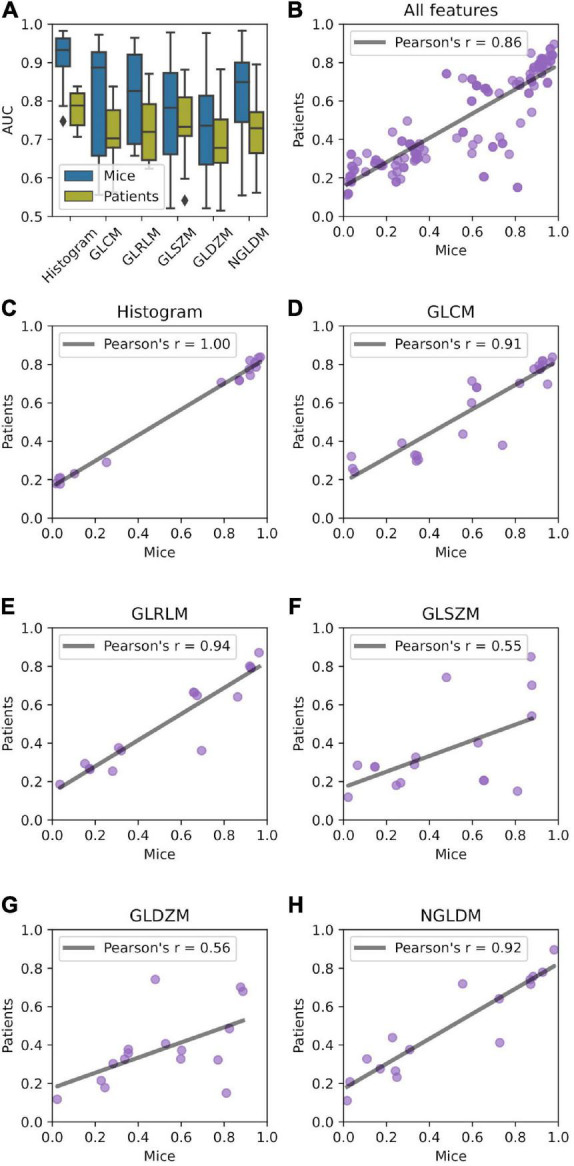
Relationship between predictive power of radiomic features in mice and patient data sets. **(A)** AUC distribution stratified by feature type (histogram, gray level co-occurrence matrix (GLCM, *n* = 26), gray level run length matrix (GLRLM, *n* = 16), gray level distance zone matrix (GLDZM, *n* = 16), gray level size zone matrix (GLSZM, *n* = 16), and neighboring gray level dependence matrix (NGLDM, *n* = 16). **(B–H)** Correlation of the AUC between mice and patient groups.

Univariate predictive power of radiomic features in terms of AUC is presented in Figure 4A. On average, features describing image intensity tended to perform better than texture-based features. Radiomic features were on average more predictive in mice than in patients. Most predictive features in mice achieved AUC = 0.988, whereas in patients AUC = 0.896. The complete list of feature predictive performance is provided in the supplement.

Univariate predictive power of the features was highly correlated between murine and patient groups (Figure 4B) with Pearson’s *r* = 0.86. Very high correlation was observed for histogram-, GLCM-, GLRLM-, and NGLDM-based features (Figures 4C–E,H). GLSZM- and GLDZM-based features exhibited more variability (Pearson’s *r* < 0.6; Figures 4F,G).

### Radiomic patterns predictive of interstitial lung disease translate from experimental interstitial lung disease to patients

To analyze transferability of radiomic patterns and models from mice to patients we built and validated four classes of models: (1) a model based on mean image intensity (MEAN), (2) a model based on first four moments of intensity distribution (mean, standard deviation, skewness, and kurtosis; MSSK), and (3) a machine learning model based on logistic regression (ML). The models were trained on mice data and tested in patients. Additionally, the models were reoptimized in patients, that is, retrained using the features from the mouse models. The results and comparison of model performance is shown in Table 2.

**TABLE 2:** Predictive performance in model tuning, testing, and re-optimization.

Model	AUC tuning (SD)	AUC testing (95% CI)	AUC re-opt. (SD)	TPR (95% CI)	TNR (95% CI)	PPV (95% CI)	NPV (95% CI)	LR + (95% CI)	LR- (95% CI)
MEAN	0.921 ± 0.048	0.774 (0.677, 0.859)	0.780 ± 0.110	0.54 (0.37, 0.71)	0.99 (0.97, 1.00)	0.96 (0.88, 1.00)	0.82 (0.73, 0.90)	53.69 (4.82, 597.44)	0.46 (0.32, 0.67)
MSSK	0.990 ± 0.017	0.815 (0.728, 0.890)	0.818 ± 0.092	0.65 (0.48, 0.82)	0.99 (0.97, 1.00)	0.97 (0.90, 1.00)	0.85 (0.77, 0.93)	64.35 (5.83, 710.65)	0.35 (0.22, 0.57)
ML	0.994 ± 0.013	0.832 (0.745, 0.907)	0.912 ± 0.058	0.78 (0.63, 0.92)	0.99 (0.97, 1.00)	0.97 (0.91, 1.00)	0.90 (0.83, 0.97)	76.92 (7.01, 844.09)	0.23 (0.12, 0.43

The uncertainty of the AUC estimates is provided as standard deviation of the cross-validation scores for model tuning and re-optimization and 95% confidence intervals for model testing. Additionally, true positive rate (TPR), true negative rate (TNR), positive predictive value (PPV), negative predictive value (NPV), positive likelihood ratio (LR +), and negative likelihood ratio (LR-) were estimated based on the cutoff point maximizing Youden’s *J* statistic (TPR + TNR-1). All 95% confidence intervals were estimated with bootstrap.

**FIGURE 5:**
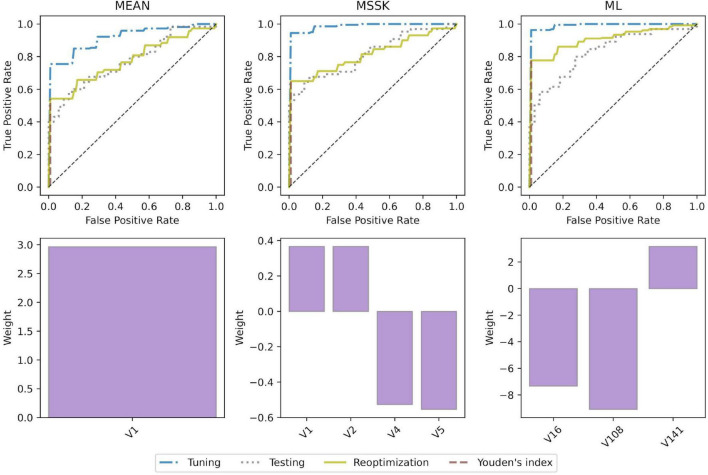
Model performance and underlying radiomic features. ROC curves and bar plots of the underlying features. V1 - mean (histogram), V2 - standard deviation (histogram), V4 - skewness (histogram), V5 - kurtosis (histogram), V16 - root mean square (histogram), V108 - gray level non-uniformity normalized (GLSZM), V141 - dependence count non-uniformity (NGLDM).

All models achieved high diagnostic performance in mice. The baseline MEAN model scored AUC = 0.921 which left little room for improvement. Nevertheless, the MSSK and the ML models exceeded AUC = 0.990 resulting in almost perfect classification performance. Testing model performance in patients resulted in AUC scores varying from 0.754 (MEAN) to 0.832 (ML). Model re-optimization in patients allowed to improve the predictive performance of all models. ROC curves associated with model tuning, testing, and re-optimization together with the underlying features are presented in Figure 5. ROC curves show that re-optimization gave little improvement for the MEAN and the MSSK models as testing and re-optimization curves followed similar characteristics. On the other hand, machine learning models improved significantly in this process. The corresponding re-optimization ROC curves detached from the testing curves to position between tuning and testing curves.

**FIGURE 6:**
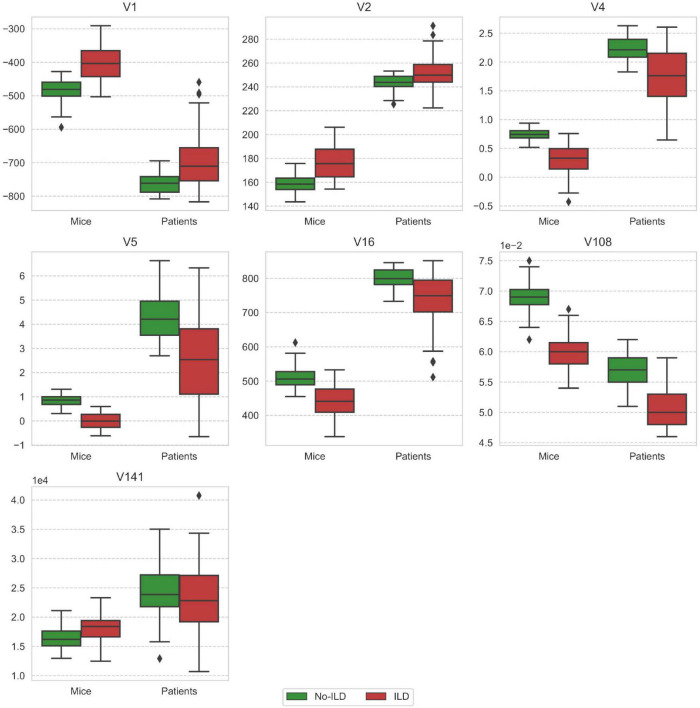
Comparison of feature distribution between mice and patient groups stratified by the ILD stage. V1 - mean (histogram), V2 - standard deviation (histogram), V4 - skewness (histogram), V5 - kurtosis (histogram), V16 - root mean square (histogram), V108 - gray level non-uniformity normalized (GLSZM), V141 - dependence count non-uniformity (NGLDM).

Substantial differences in distribution of radiomic features included in the models in terms of location and dispersion are presented in Figure 6. Most of the features exhibit patterns of the same direction in both mice and patient data sets, that is, either rising or falling trend from healthy to ILD.

## Discussion

In this analysis, we report that radiomic features and models can be translated from experimental to human ILD. Collectively, our data suggest that well characterized and representative animal models could represent valuable systems for defined hypothesis testing in radiomics research, particularly for evaluating links with pathophysiology or studying responses to targeted therapies in rare diseases with low number of patients and limited access to tissue samples.

Radiomic features proved to be highly indicative of experimental- and SSc-ILD. Furthermore, we observed strong linear correlation in terms of discriminative power between features extracted from mice microCT scans and patient HRCT. We also showed that multivariate models of ILD translated well from mice to patient data sets. Nevertheless, we observed the differences between the data sets in terms of feature classes that were predictive. In mice, most of the feature groups contained features that reached similar maximum AUC scores. On the other hand, in patients we observed that even though histogram-based features achieved high discriminative power, some texture features were more predictive. This difference could be caused by inferior quality of microCT compared to HRCT. For this reason, the assessment of microCT done by our radiologist might have also been mainly led by first order characteristics rather than texture. Furthermore, the ILD manifestations can differ depending on the etiology. As a result, the observed differences may be caused by the limitation of the bleomycin-induced ILD being an imperfect model of SSc-ILD. In any case, our results are in line with the available literature on human lung pathologies including chronic obstructive pulmonary disease, radiation-induced pneumonitis or connective tissue disease-related ILD, which showed that texture-based analysis of CT data can be superior compared to the visual or histogram-based measures for diagnosis (28, 31, 32).

Analysis of feature weights in the MEAN and the MSSK models showed that higher values of the mean and standard deviation of the image intensity and lower values of skewness and kurtosis correspond to larger risk of ILD. Effectively, this means that presence of ILD shifts the intensity distribution from a typical “healthy” positively skewed intensity distribution toward higher intensity values with a more symmetric distribution and thin tails. The best performing model (ML) relied on three radiomic features: the root mean square (histogram), gray level non-uniformity normalized (GLSZM), and dependence count non-uniformity (NGLDM). Significant improvement of machine learning models by re-optimization may suggest the existence of similar predictive radiomic patterns in training (mice) and test (patients) data sets in presence of domain shift between both groups.

The presented study has a few limitations. First, the differences in scanning parameters between microCT and HRCT cause a significant domain shift between experimental and patient data sets. Although, we were able to recover the predictive power of the analyzed multivariate models by re-optimization in the patient cohort, and by that confirm transferability of the underlying radiomic signatures, better calibration of the microCT scanner and selection of scanning parameters could potentially improve the transferability. Second, our study focused on CT-derived radiomics approaches, since HRCT scans are part of the routine work-up of ILD patients. Other imaging modalities such as nuclear imaging or MRI, although currently rarely performed in ILD (10), could be evaluated for radiomic analyses to assess whether they might provide additional or complementary information.

## Conclusion

Radiomic signatures of experimental ILD derived from microCT scans translated as prognostic factors to HRCT of SSc-ILD. By this we showed that the well-established experimental model of BLM-induced ILD is a valuable system to test defined hypotheses in radiomics research for later validation in human cohorts.

## Data availability statement

The raw data supporting the conclusions of this article will be made available by the authors, without undue reservation.

## Ethics statement

The studies involving human participants were reviewed and approved by the local ethics committees (approval numbers: pre-BASEC-EK-839 (KEK-no.-2016-01515), KEK-ZH-no. 2010-158/5, BASEC-no. 2018-02165, and BASEC-no. 2018-01873). The patients/participants provided their written informed consent to participate in this study. This animal study was reviewed and approved by the cantonal authorities and performed in compliance with the Swiss law of animal protection (ZH235-2018).

## Author contributions

HG, JG-S, MG, BM, and ST-L contributed to the conception and design of the study. HG, JG-S, MBr, CB, and TF contributed to the acquisition and analysis of the data. HG, JG-S, BM, and ST-L contributed to the interpretation of data. HG and MBo contributed to the creation of software used in the study. All authors have drafted the work or substantively revised it.

## References

[1] Hutchinson JP McKeever TM Fogarty AW Navaratnam V Hubbard RB. Increasing global mortality from idiopathic pulmonary fibrosis in the twenty-first century. *Ann Am Thorac Soc.* (2014) 11:1176–85. 10.1513/AnnalsATS.201404-145OC 25165873

[2] Wallace B Vummidi D Khanna D. Management of connective tissue diseases associated interstitial lung disease: a review of the published literature. *Curr Opin Rheumatol.* (2016) 28:236–45. 27027811 10.1097/BOR.0000000000000270 PMC4826478

[3] John Gibson G Loddenkemper R Sibille Y Lundbäck B. *The European Lung White Book: Respiratory Health and Disease in Europe.* Lausanne: European Respiratory Society (2013). 10.1183/09031936.00105513 24000245

[4] Carrington R Jordan S Pitchford SC Page CP. Use of animal models in IPF research. *Pulm Pharmacol Ther.* (2018) 51:73–8. 29981850 10.1016/j.pupt.2018.07.002

[5] Tashiro J Rubio GA Limper AH Williams K Elliot SJ Ninou I Exploring animal models that resemble idiopathic pulmonary fibrosis. *Front Med.* (2017) 4:118. 10.3389/fmed.2017.00118 28804709 PMC5532376

[6] Schniering J Guo L Brunner M Schibli R Ye S Distler O Evaluation of Tc-rhAnnexin V-128 SPECT/CT as a diagnostic tool for early stages of interstitial lung disease associated with systemic sclerosis. *Arthritis Res Ther.* (2018) 20:183. 10.1186/s13075-018-1681-1 30115119 PMC6097327

[7] Zhou Y Chen H Ambalavanan N Liu G Antony VB Ding Q Noninvasive imaging of experimental lung fibrosis. *Am J Respir Cell Mol Biol.* (2015) 53:8–13. 25679265 10.1165/rcmb.2015-0032TR PMC4566116

[8] Silver KC Silver RM. Management of systemic-sclerosis-associated interstitial lung disease. *Rheum Dis Clin North Am.* (2015) 41:439–57. 26210128 10.1016/j.rdc.2015.04.006 PMC4515778

[9] Collins BF Raghu G Idiopathic pulmonary fibrosis: How should a confident diagnosis be made? In: Thillai M Moller DR Meyer KC editors. *Clinical Handbook of Interstitial Lung Disease*. (Boca Raton, FL: CRC Press) (2017). p. 135–48.

[10] Hansell DM Goldin JG King TE Lynch DA Richeldi L Wells AU. CT staging and monitoring of fibrotic interstitial lung diseases in clinical practice and treatment trials: a position paper from the fleischner society. *Lancet Respirat Med.* (2015) 3:483–96. 10.1016/S2213-2600(15)00096-X 25975761

[11] Wells AU Desai SR Rubens MB Goh NSL Cramer D Nicholson AG Idiopathic pulmonary fibrosis: a composite physiologic index derived from disease extent observed by computed tomography. *Am J Respir Crit Care Med.* (2003) 167:962–9. 10.1164/rccm.2111053 12663338

[12] Aichler M Kunzke T Buck A Sun N Ackermann M Jonigk D Molecular similarities and differences from human pulmonary fibrosis and corresponding mouse model: MALDI imaging mass spectrometry in comparative medicine. *Lab Invest.* (2018) 98:141–9. 10.1038/labinvest.2017.110 29035378

[13] Schniering J Benešová M Brunner M Haller S Cohrs S Frauenfelder T F-AzaFol for detection of folate receptor-β positive macrophages in experimental interstitial lung disease-a proof-of-concept study. *Front Immunol.* (2019) 10:2724. 10.3389/fimmu.2019.02724 31824505 PMC6883947

[14] Schniering J Benešová M Brunner M Haller S Cohrs S Frauenfelder T Visualisation of interstitial lung disease by molecular imaging of integrin αvβ3 and somatostatin receptor 2. *Ann Rheum Dis.* (2019) 78:218–27. 30448769 10.1136/annrheumdis-2018-214322

[15] Schniering J Gabrys H Brunner M Distler O Guckenberger M Bogowicz M Computed-tomography-based radiomics features for staging of interstitial lung disease – transferability from experimental to human lung fibrosis - a proof-of-concept studyImaging. *Eur Respir Soc.* (2019) 54:PA4806. 10.1183/13993003.congress-2019.pa4806

[16] Lambin P Rios-Velazquez E Leijenaar R Carvalho S van Stiphout RGPM Granton P Radiomics: extracting more information from medical images using advanced feature analysis. *Eur J Cancer.* (2012) 48:441–6. 22257792 10.1016/j.ejca.2011.11.036 PMC4533986

[17] Lambin P Leijenaar RTH Deist TM Peerlings J de Jong EEC van Timmeren J Radiomics: the bridge between medical imaging and personalized medicine. *Nat Rev Clin Oncol.* (2017) 14:749–62. 10.1038/nrclinonc.2017.141 28975929

[18] Aerts HJWL Velazquez ER Leijenaar RTH Parmar C Grossmann P Carvalho S Decoding tumour phenotype by noninvasive imaging using a quantitative radiomics approach. *Nat Commun.* (2014) 5:4006. 10.1038/ncomms5006 PMC4059926 24892406

[19] Lu H Arshad M Thornton A Avesani G Cunnea P Curry E A mathematical-descriptor of tumor-mesoscopic-structure from computed-tomography images annotates prognostic- and molecular-phenotypes of epithelial ovarian cancer. *Nat Commun.* (2019) 10:764. 10.1038/s41467-019-08718-9 30770825 PMC6377605

[20] Wu W Parmar C Grossmann P Quackenbush J Lambin P Bussink J Exploratory study to identify radiomics classifiers for lung cancer histology. *Front Oncol.* (2016) 6:71. 10.3389/fonc.2016.00071 27064691 PMC4811956

[21] Grossmann P Stringfield O El-Hachem N Bui MM Rios Velazquez E Parmar C Defining the biological basis of radiomic phenotypes in lung cancer. *Elife.* (2017) 6:e23421. 10.7554/eLife.23421 28731408 PMC5590809

[22] Parmar C Leijenaar RTH Grossmann P Rios Velazquez E Bussink J Rietveld D Radiomic feature clusters and prognostic signatures specific for lung and head & neck cancer. *Sci Rep.* (2015) 5:11044. 10.1038/srep11044 26251068 PMC4937496

[23] Leijenaar RT Bogowicz M Jochems A Hoebers FJ Wesseling FW Huang SH Development and validation of a radiomic signature to predict HPV (p16) status from standard CT imaging: a multicenter study. *Br J Radiol.* (2018) 91:20170498. 10.1259/bjr.20170498 29451412 PMC6223271

[24] Aerts HJWL Grossmann P Tan Y Oxnard GR Rizvi N Schwartz LH Defining a Radiomic response phenotype: a pilot study using targeted therapy in NSCLC. *Sci Rep.* (2016) 6:33860. 10.1038/srep33860 PMC5028716 27645803

[25] Schniering J Maciukiewicz M Gabrys HS Brunner M Blüthgen C Meier C Computed tomography-based radiomics decodes prognostic and molecular differences in interstitial lung disease related to systemic sclerosis. *Eur Respir J.* (2021) 59:2004503. 10.1183/13993003.04503-2020 34649979 PMC9117734

[26] Walsh SLF Calandriello L Silva M Sverzellati N. Deep learning for classifying fibrotic lung disease on high-resolution computed tomography: a case-cohort study. *Lancet Respir Med.* (2018) 6:837–45. 10.1016/S2213-2600(18)30286-8 30232049

[27] Humphries SM Yagihashi K Huckleberry J Rho B-H Schroeder JD Strand M Idiopathic pulmonary fibrosis: data-driven textural analysis of extent of fibrosis at baseline and 15-month follow-up. *Radiology.* (2017) 285:270–8. 10.1148/radiol.2017161177 28493789 PMC5621716

[28] Kloth C Blum AC Thaiss WM Preibsch H Ditt H Grimmer R Differences in texture analysis parameters between active alveolitis and lung fibrosis in chest CT of patients with systemic sclerosis: a feasibility study. *Acad Radiol.* (2017) 24:1596–603. 10.1016/j.acra.2017.07.002 28807589

[29] Kloth C Henes J Xenitidis T Thaiss WM Blum AC Fritz J Chest CT texture analysis for response assessment in systemic sclerosis. *Eur J Radiol.* (2018) 101:50–8. 10.1016/j.ejrad.2018.01.024 29571801

[30] Lee SM Seo JB Oh SY Kim TH Song JW Lee SM Prediction of survival by texture-based automated quantitative assessment of regional disease patterns on CT in idiopathic pulmonary fibrosis. *Eur Radiol.* (2018) 28:1293–300. 28929225 10.1007/s00330-017-5028-0

[31] Sorensen L Nielsen M Lo P Ashraf H Pedersen JH de Bruijne M. Texture-based analysis of COPD: a data-driven approach. *IEEE Trans Med Imaging.* (2012) 31:70–8. 10.1109/TMI.2011.2164931 21859615

[32] Cunliffe AR Armato SG III Straus C Malik R Al-Hallaq HA. Lung texture in serial thoracic CT scans: correlation with radiologist-defined severity of acute changes following radiation therapy. *Phys Med Biol.* (2014) 59:5387–98. 10.1088/0031-9155/59/18/5387 25157625 PMC4199222

[33] Eresen A Yang J Shangguan J Li Y Hu S Sun C MRI radiomics for early prediction of response to vaccine therapy in a transgenic mouse model of pancreatic ductal adenocarcinoma. *J Transl Med.* (2020) 18:61. 32039734 10.1186/s12967-020-02246-7 PMC7011246

[34] Eresen A Yang J Shangguan J Benson AB Yaghmai V Zhang Z. Detection of immunotherapeutic response in a transgenic mouse model of pancreatic ductal adenocarcinoma using multiparametric MRI radiomics: a preliminary investigation. *Acad Radiol.* (2021) 28:e147–54. 10.1016/j.acra.2020.04.026 32499156 PMC7704817

[35] Núñez LM Romero E Julià-Sapé M Ledesma-Carbayo MJ Santos A Arús C Unraveling response to temozolomide in preclinical GL261 glioblastoma with MRI/MRSI using radiomics and signal source extraction. *Sci Rep.* (2020) 10:19699. 33184423 10.1038/s41598-020-76686-y PMC7661707

[36] Becker AS Schneider MA Wurnig MC Wagner M Clavien PA Boss A. Radiomics of liver MRI predict metastases in mice. *Eur Radiol Exp.* (2018) 2:11. 10.1186/s41747-018-0044-7 29882527 PMC5971192

[37] Ni M Wang L Yu H Wen X Yang Y Liu G Radiomics approaches for predicting liver fibrosis with nonenhanced T1 -weighted imaging: comparison of different radiomics models. *J Magn Reson Imaging.* (2021) 53:1080–9. 33043991 10.1002/jmri.27391

[38] Schniering J Borgna F Siwowska K Benešová M Cohrs S Hasler R In vivo labeling of plasma proteins for imaging of enhanced vascular permeability in the lungs. *Mol Pharm.* (2018) 15:4995–5004. 30265552 10.1021/acs.molpharmaceut.8b00606

[39] Minier T Guiducci S Bellando-Randone S Bruni C Lepri G Czirják L EUSTAR co-workers, Preliminary analysis of the very early diagnosis of systemic sclerosis (VEDOSS) EUSTAR multicentre study: evidence for puffy fingers as a pivotal sign for suspicion of systemic sclerosis. *Ann Rheum Dis.* (2014) 73:2087–93. 23940211 10.1136/annrheumdis-2013-203716

[40] van den Hoogen F Khanna D Fransen J Johnson SR Baron M Tyndall A 2013 classification criteria for systemic sclerosis: an American college of rheumatology/European league against rheumatism collaborative initiative. *Arthritis Rheum.* (2013) 65:2737–47. 24122180 10.1002/art.38098 PMC3930146

[41] Zwanenburg A Leger S Vallières M Löck S. Image biomarker standardisation initiative. *arXiv* [Preprint] (2016). arXiv:1612.07003 [cs.CV].

[42] Shrout PE Fleiss JL. Intraclass correlations: uses in assessing rater reliability. *Psychol Bull.* (1979) 86:420–8. 18839484 10.1037//0033-2909.86.2.420

[43] Hunter JD Matplotlib: A 2D graphics environment. *Comput Sci Eng*. (2007) 9:99–104.

[44] Van Der Walt S Colbert SC Varoquaux G The NumPy array: A structure for efficient numerical computation. *Comput Sci Eng*. (2011) 13:22–30.

[45] McKinney W Data structures for statistical computing in python. In: *Proceedings of the 9th Python in Science Conference*. Austin, TX (2010). p. 51–6.

[46] Pedregosa F Varoquaux G Gramfort A Michel V Thirion B Grisel O Scikit-learn: Machine learning in python. *J Mach Learn Res*. (2011) 12:2825–30.

